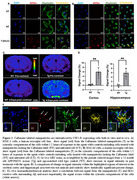# Magnetic resonance molecular imaging of neuroinflammation in mouse models of Alzheimer's and Parkinson's diseases by precision targeting of CSF1‐R

**DOI:** 10.1002/alz70856_107465

**Published:** 2026-01-09

**Authors:** Xianwei Sun, Andrew A Badachhape, Terry‐Elinor Reid, Jeannie Chin, Joshua M Shulman, Ananth Annapragada, Henry Lowe, Ngeh Toyang, Eric Tanifum

**Affiliations:** ^1^ Baylor College of Medicine, Houston, TX, USA; ^2^ Concordia University of Wisconsin, Mequon, WI, USA; ^3^ Jan and Dan Duncan Neurological Research Institute, Texas Children's Hospital, Houston, TX, USA; ^4^ Texas Children's Hospital/Baylor College of Medicine, Houston, TX, USA; ^5^ Flavocure Biotech, Inc, Baltimore, MD, USA

## Abstract

**Background:**

Microglia‐mediated neuroinflammation plays a pivotal role in the initiation and propagation of pathological markers of neurodegenerative disorders including Alzheimer's disease (AD) and Parkinson's disease (PD). CSF1‐R signaling mediates microglial activation, proliferation, and survival; both CSF1‐R upregulation and increased proliferation of microglia have been observed in AD patients. Imaging technologies that effectively profile reactive microgliosis in vivo have the potential to be diagnostic tools, facilitate patient stratification in clinical trials, and monitor treatment. There is currently no clinically approved tool to profile reactive microgliosis *in vivo*. We present a novel molecular imaging technique for neuroinflammation by targeting CSF1‐R with MRI sensitive liposomes.

**Method:**

Liposomes with DSPE‐PEG‐Caflanone as the targeting moiety, and Gd(III) DSPE‐DOTA as the MRI contrast source were formulated using standard protocols. Caflanone is a potent CSF1‐R ligand. In vitro nanoparticle cell uptake studies and blocking experiments with free Caflanone employing both human and mouse microglia cell lines were used to establish receptor‐mediated internalization of the agent. Two AD mouse models (APP/PSEN1 and P301S) and one PD mouse model (A53TαS Tg) were used in *in vivo* MRI studies. Controls included wild type (WT) litter mates injected with the same agent. Mice were pre‐scanned to establish a baseline followed by injection of the agent. Post‐contrast scans were obtained at 4 days post‐injection, followed by euthanasia, and brain removal for ex vivo immunohistochemical analysis.

**Result:**

As shown in Figure 1, in vitro cell assays showed internalization of the Caflanone labeled nanoparticles within 1.5 hours of exposure. Coincubation with increasing concentrations of free Caflanone resulted in diminished particle uptake at high concentrations of the free ligand. *In vivo* MRI demonstrated increased brain retention of the nanoparticles in transgenic mice compared to controls. Ex‐vivo immunohistochemical analysis showed the nanoparticles in the cytosolic compartment of IBA1 reactive cells surrounding amyloid‐β (Aβ) plaques in the APP/PSEN1 mouse model.

**Conclusion:**

Results demonstrate precision delivery of Caflanone labeled liposomes bearing an MRI diagnostic payload to activated microglia in three different mouse models of neurodegenerative disorders. These nanoparticles can also deliver a therapeutic payload with similar precision, opening a new frontier for the development of both diagnostics and therapeutics for neuroinflammation.